# Expectancy Effects Threaten the Inferential Validity of Synchrony-Prosociality Research

**DOI:** 10.1162/opmi_a_00067

**Published:** 2022-12-16

**Authors:** S. Atwood, Adena Schachner, Samuel A. Mehr

**Affiliations:** Department of Psychology, Princeton University, Princeton, NJ 08540 USA; Department of Psychology, University of California San Diego, La Jolla, CA 92093-0109 USA; Haskins Laboratories, Yale University, New Haven, CT 06511 USA; School of Psychology, University of Auckland, Auckland 1010, New Zealand

**Keywords:** synchrony, prosociality, expectancy, experimenter bias, placebo effects

## Abstract

Many studies argue that synchronized movement increases prosocial attitudes and behavior. We reviewed meta-analytic evidence that reported effects of synchrony may be driven by experimenter expectancy, leading to experimenter bias; and participant expectancy, otherwise known as placebo effects. We found that a majority of published studies do not adequately control for experimenter bias and that multiple independent replication attempts with added controls have failed to find the original effects. In a preregistered experiment, we measured participant expectancy directly, asking whether participants have *a priori* expectations about synchrony and prosociality that match the findings in published literature. Expectations about the effects of synchrony on prosocial attitudes directly mirrored previous experimental findings (including both positive and null effects)—despite the participants not actually engaging in synchrony. On the basis of this evidence, we propose an alternative account of the reported bottom-up effects of synchrony on prosociality: the effects of synchrony on prosociality may be explicable as the result of top-down expectations invoked by placebo and experimenter effects.

## INTRODUCTION

From the sympathetic oscillation of clocks (Huygens, 1665) to the flight patterns of migratory birds (Gunnarsson et al., 2004), synchrony is a widespread element of our physical, biological, and social worlds (Arenas et al., 2008; Bernieri & Rosenthal, 1991; Osipov et al., 2007; Phillips-Silver et al., 2010; Pikovsky et al., 2003). In humans, synchronous behavior is universal (in the form of dance; Mehr et al., 2018, 2019) and sweeping claims about the importance of synchrony to human social life, human cognition, and human evolution are common (e.g., Clarke et al., 2015; Launay et al., 2016; McNeill, 1995 and many others; for discussion, see Mehr et al., 2021).

Many have argued that synchronized movement facilitates human social interaction, aligning emotional and mental states and promoting social cohesion (Dissanayake, 2000; Durkheim, 1912; McNeill, 1995; Tarr et al., 2016; Turner, 1995). More recently, studies have found empirical evidence of synchrony’s positive effects on prosociality: Synchrony is reported to promote cooperation (Reddish et al., 2013; Valdesolo et al., 2010; Wiltermuth & Heath, 2009), affiliative behaviors (Macrae et al., 2008; Paladino et al., 2010), positive emotions (Hove & Risen, 2009; Launay et al., 2014), connection (Hove, 2008; Lakens, 2010; Wiltermuth & Heath, 2009), trust (Launay et al., 2013), and compassion for others (Valdesolo & DeSteno, 2011).

These findings support theoretical arguments that synchrony plays a causal role in the evolution of human social groups and the structure of human society (Clarke et al., 2015; Jackson et al., 2018; Lakens, 2010; Launay et al., 2016; Loersch & Arbuckle, 2013; Pearce et al., 2015; Savage et al., 2021; Weinstein et al., 2016). These in turn have been applied in real-world settings to ameliorate intergroup conflict (Tunçgenç & Cohen, 2016) and to design social robots (Delaherche et al., 2012).

Importantly, these claims go beyond the related idea that imitation, coordinated movements, and shared goals—independent of synchrony—increase prosociality, for which other literatures provide evidence (e.g., Carpenter et al., 2013; Chartrand & van Baaren, 2009; Over & Carpenter, 2015; Sherif, 1958). Indeed, recent work on synchrony aimed to control for these factors as potential confounds (e.g., Reddish et al., 2013; Tarr et al., 2018). The key claim is that synchronized movement is reliably more effective at promoting prosociality than is movement that is not synchronized (even if it is coordinated), as a result of bottom-up alignment of motoric/visual input, with aligned sensory input increasing merging of representations of self and other (e.g., Clarke et al., 2015; Tarr et al., 2014).

Here, we examine an alternative account for such claims. Meta-analytic evidence suggests that the effects of synchrony on prosociality may be driven by expectancy effects generated by a combination of (1) experimenter expectancy, leading to experimenter bias; and (2) participant expectancy (i.e., placebo effects). Rather than a result of bottom-up alignment of motoric and visual input, or “self-other merging” (e.g., Clarke et al., 2015; Tarr et al., 2014), we argue that the link between synchrony and prosociality is more parsimoniously explained as the result of top-down expectations. We evaluate the evidence for this claim in published studies, report new evidence demonstrating expectancy in real participants, and suggest ways future work can tease apart effects of synchrony from effects of expectancy.

### Evidence of Experimenter Bias in the Synchrony Literature

*Experimenter bias* occurs when differences in an experimenter’s behavior shape participant response to align with experimenters’ expectations (Harris & Rosenthal, 1985; Rosenthal, 1976). These are most evident in study designs where both participants and experimenters are aware of their assigned experimental condition (Rosenthal, 1976). When experimenters are not naïve to experimental condition, biases can be large and are common across many study situations, even if experimenter-participant contact is limited. Rosenthal and Rubin (1978) estimated the overall size of interpersonal expectancy effects (including those caused by experimenter biases) across 345 studies in eight domains at *d* = .70.

Most published studies on synchrony and prosocial behavior involve experimenters who were aware of participants’ condition during the study (estimated at 75%; where experimenters were categorized as naïve if the authors stated explicitly that the experimenter was not aware of the hypotheses or condition, or was not present during the study; Rennung & Göritz, 2016), raising the possibility that differences in the ways experimenters interacted with participants unconsciously shaped their responses to align with experimenters’ expectations (Rosenthal, 1976). Two meta-analyses found weak-to-moderate effects of interpersonal synchrony on prosociality (Mogan et al., 2017; Rennung & Göritz, 2016), but when limiting the meta-analysis to experiments where the experimenter was naïve to condition, the effect of synchrony on prosocial behavior was not distinguishable from zero (Rennung & Göritz, 2016). Thus, methodological choices, such as allowing experimenters to be aware of participants’ condition assignment, or unequal degrees of participant expectancy, may leave synchrony-prosociality literature vulnerable to the role of bias via experimenter demand effects (Rennung & Göritz, 2016).

In line with this meta-analytic finding, multiple attempts to replicate the effects of highly-cited studies in the synchrony literature with added controls for experimenter bias have failed (Kirschner & Ilari, 2013; Schachner & Garvin, 2010). For example, Schachner and Garvin (2010) conducted two replications of the synchrony manipulation used in Wiltermuth and Heath (2009; Exp. 3) with experimenters naïve to condition and found that, unlike the original study, synchrony did not increase interpersonal cooperation, conformity, trust, similarity, or feelings of being in the same team. Additionally, other studies found no prosocial effects of synchrony in young children (Kirschner & Ilari, 2013).

### The Potential for Placebo Effects Caused by Participant Expectancy

A *placebo effect* occurs when a response is misattributed to an inert treatment or experimental condition, but is actually due to contextual factors such as pre-existing beliefs (Benedetti et al., 2005). Placebo effects exist across many clinical studies (see reviews: Finniss et al., 2010; Kaptchuk & Miller, 2015; Price et al., 2008), including those in psychology (e.g., Kirsch & Sapirstein, 1998; Leuchter et al., 2002). While the psychological mechanisms underlying placebo effects are controversial (e.g., Moerman, 2002; Moerman & Jonas, 2002; Stewart-Williams & Podd, 2004), their results are clear: When people expect that a particular intervention will lead to a particular effect, this expectation alone can cause them to experience that effect (Hahn, 1997; Kirsch, 1985, 1990; Montgomery & Kirsch, 1997; Peck & Coleman, 1991).

*Participant expectancy effects* are a well-documented form of placebo effects in the clinical and experimental psychology literature (Chan & Lovibond, 1996; Colagiuri, 2010; Goldstein, 2013; Oettingen, 2000; Younger et al., 2012). For example, participant expectancy affects memory for previous experiences, biasing subjective outcome measures in favor of an intervention (Price et al., 1999). Moreover, randomized-controlled trials investigating the role of placebo effects find that participants in pain studies who expect a particular treatment to be effective (e.g., acupuncture) report significantly less pain, irrespective of whether they experienced actual acupuncture, or a control treatment (Linde et al., 2007). Recent work has also found that highly structured interventions lead to stronger placebo effects than less-structured interventions (Shen et al., 2020).

The elimination of bias introduced by expectation-based placebo effects is an essential step to ensure the validity of findings in psychological science—yet is often overlooked. In many studies, intervention groups are compared to control groups without accounting for differences in expectations (Boot et al., 2013). In the case of motor synchrony, expectation-based effects may arise due to explicit instruction (e.g., from an experimenter, to “walk in step” as in Wiltermuth & Heath, 2009). However, they may also arise incidentally: By observing their own participation in the task, participants become aware of the level of synchrony involved, and rate the level of synchrony as different across conditions (e.g., Tarr et al., 2018). Placebo effects therefore remain a potential concern even in tasks where synchrony arises without explicit instruction. Regardless of origin, participants’ *a priori* expectations about the heightened effects of an experimental manipulation on a dependent variable may be attributable to placebo rather than to the manipulation itself. Thus, comparison to an active control that is matched for participant expectations is necessary to fully attribute effects to the intervention, rather than participant expectancy (Boot et al., 2013).

Do participants in synchrony experiments enter with *a priori* expectations about the relationship between synchrony and prosociality? If so, this would leave the synchrony literature vulnerable to placebo effects, and imply that participant expectancy, rather than the experience of synchrony itself, may be responsible for increases in prosociality after synchrony. If true, the bottom-up effects of synchronized behavior may be small or nonexistent, and top-down expectations would the primary driver of synchrony’s prosocial effects.

### Do Participants Have *A Priori* Expectations about Synchrony and Prosociality?

To test this question, we conducted a preregistered experiment (see https://osf.io/6g2vy) to measure the degree to which participants have *a priori* expectations about synchrony and prosociality, and whether these expectations match the findings reported in the literature (see full methods details in Supplementary Information Text S1–S3). Participants read about a hypothetical experiment (Exp. 1 in Wiltermuth and Heath (2009), among the most-cited synchrony papers in the literature), and were asked to predict the attitudes and emotions others would have after participating. In the original experiment, participants took a 7-minute walk around a college campus together with two other participants and an experimenter. Participants in the experimental condition were instructed to walk in synchrony, while participants in the control condition were not (Wiltermuth & Heath, 2009; S. Wiltermuth, personal communication). Afterward, participants were surveyed regarding their attitudes toward the other participants and their emotions, including the extent of interpersonal coordination, connection, trust, similarity, synchrony, feeling of being on the same team, happiness, and frustration. Participants in the synchrony condition showed greater levels of connection, trust, same team feeling, and feelings of similarity to their partner in comparison to participants in the control condition; but did not differ on levels of happiness (Wiltermuth & Heath, 2009).

We asked participants (*N* = 216) to imagine this hypothetical experiment, and probed their expectations by asking them to predict how other people would feel and act after participating in such a task, using measures that mirrored those of the original study (provided by the authors; S. Wiltermuth, personal communication). We then compared our participants’ expectations to the real effects (both positive and null) reported by Wiltermuth and Heath (2009). We reasoned that if participants in our hypothetical scenario study showed the same distinct pattern of positive and null expectations as participants in the original study showed after actually experiencing synchrony or asynchrony, then participant expectancy is likely to play a role in the original experimental findings. To isolate participant expectancy from the effects of first-person mental simulation of synchrony (see Atherton et al., 2019; Stupacher et al., 2017), we asked participants to make predictions about other people, not themselves; and minimized vivid imagery by keeping vignettes brief and not prompting participants to take any additional time elaborating on what they read (as done in Atherton et al., 2019). We note that while such measures do not preclude the possibility of *all* mental simulation, it is likely that participants in our study engaged in minimal mental simulation as a result of these aspects of the study design.

## RESULTS

**Figure 1.**
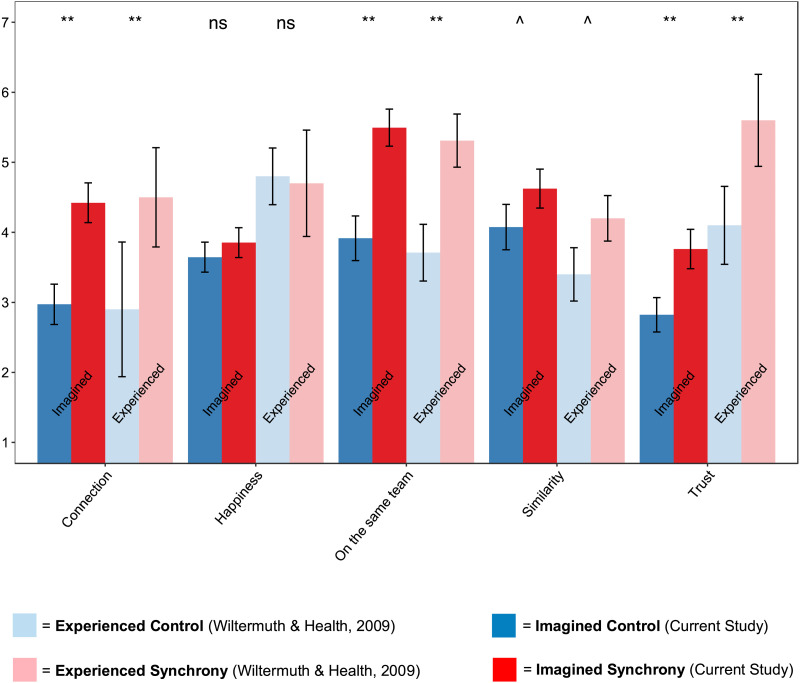
**Participants’ expectations about synchrony match reported effects of the experience of synchrony, from a previous study.** Each set of bars represents participants’ ratings of emotions and attitudes toward other people in one of five different categories (*x-axis*) after *actually* walking with them in synchrony (Experienced Synchrony; data from Wiltermuth & Heath, 2009; Exp. 1); *actually* walking normally/out of synchrony (Experienced Control; data from Wiltermuth & Heath, 2009; Exp. 1); *imagining* how other people would expect to feel after walking in synchrony (Imagined Synchrony); or *imagining* how others would feel after walking normally (Imagined Control). Error bars represent 95% confidence intervals. **p < 0.00625, ˆ*p* < 0.05.

Participants’ expectations about the effects of synchrony tightly matched the effects of the first-person experience of synchrony (see Figure 1): Participants predicting people’s attitudes after walking in synchrony (versus the control condition) predicted that they would feel more connected, trusting, more on the same team (*p*s < .00625, the Bonferroni-adjusted alpha for 8 comparisons), and more similar to their group members (*p* < .05; this test did not survive the correction for multiple comparisons). Notably, participants did *not* expect differences in levels of happiness (*p* = .17), mirroring the null result reported in Wiltermuth and Heath (2009; Exp. 1). Participants in our study also expected people to feel more coordinated and synchronized (*p*’s < .00625), but not more frustrated (*p* > .78) after walking in synchrony (see Supplement Table S1 and Figure S2 for means, standard deviations, and visualization of all comparisons).

On the suggestion of an anonymous reviewer, we continued by testing whether the expectancy effects reported above differ in magnitude from the effects in Exp. 1 of Wiltermuth and Heath (2009; many thanks to Scott Wiltermuth for providing the original data to enable this analysis). We ran linear regressions predicting each variable tested in both samples (connection, trust, similarity, synchrony, feeling of being on the same team, and happiness) from the predictors of study type (imagined, in the current study; vs. experienced, in the Wiltermuth & Heath study), condition (synchrony vs. control/neutral), and their interaction.

We found no evidence of differences in magnitude of the main effects across the two study types for connection, trust, similarity, same team, or happiness; and interactions between study type and condition were also not significant (all *p*s > .1). There was a difference by study type only for the feeling of being in synchrony, such that people who experienced synchrony (relative to those who imagined it in others) showed a greater difference between synchrony vs. control condition in how synchronized they felt (or imagined others felt) with their partner (*β* = 1.52, *t*(242) = 2.75, *p* = 0.006. The full regression results are reported in Appendix S3 in the Supplementary Information.

## DISCUSSION

Effects of synchrony on prosociality are typically attributed to the experience of synchrony, with first-person sensory experience of moving in synchrony with another person causing increased prosociality. Based on patterns of reproducibility and the results outlined above, we suspect an alternative: Synchrony may lead to prosocial effects not because of experiencing the content of the manipulation, but rather, due to systematic *a priori* expectations about the effects of synchrony, held by both participants and experimenters.

We are not the first to address the role of expectancy in the synchrony literature. Past work raised the possibility of a moderating role for experimenter effects, in that experimental designs that did control for experimenter effects (of which there were fairly few) showed small to null effects of interpersonal synchrony on prosocial attitudes and behaviors (see Rennung & Göritz, 2016). While this conclusion is not necessarily reflective of Rennung & Goritz’s interpretation, these data are consistent with the idea that expectancy in general inflates effect sizes (Price et al., 2008; Rosenthal & Rubin, 1978). The data presented here take these ideas one step further: we provide an existence proof that expectancy regarding prosocial attitudes can be elicited in studies of synchrony, supporting the idea of a generalized, top-down causal pathway for effects of synchrony on prosociality that may be overlooked in favor of bottom-up perceptual explanations.

Neither the existing literature nor our survey demonstrate an absence of a link between synchrony and prosociality. Experimenter bias and placebo effects may only account for a fraction of a true effect of synchrony on prosociality, and may apply differently depending on the type of movement (finger tapping vs. walking) or social context (in virtual reality vs. walking around campus). Still, given the propensity of expectations to produce large effects, their effects are likely to be substantial. Understanding the role of expectancy is therefore essential to understanding the psychological mechanisms underlying synchrony’s effects. Coordinated behaviors such as imitation or having a shared goal are also thought to increase prosociality, whether or not they involve synchronized movement (e.g., Carpenter et al., 2013; Chartrand & van Baaren, 2009; Over & Carpenter, 2015; Sherif, 1958). Thus, even if differences between synchrony and asynchrony are attributable to expectancy, activities that promote coordinated behavior (such as music and dance) could be expected to promote prosociality, independent of putative effects of synchrony.

Where might people’s expectations about synchrony come from? One possibility is that they reflect accurate metacognition: People may be aware of the impact of synchrony on their attitudes, emotions, and behavior. We find such nuanced metacognition unlikely; however, even accurate expectations would create placebo effects. In this case, placebo effects would systematically inflate the effect size of the experience of synchrony in the literature.

An alternative possibility is that people have intuitive beliefs about synchrony as a part of their folk theories of social behavior. From childhood, humans develop intuitive mental theories of how the social and physical worlds work. These are shaped both by experience and by innate biases about how people learn and think (Gopnik & Meltzoff, 1997; Spelke & Kinzler, 2007). While rich, these intuitive theories are often wrong (Gelman, 2003; Shtulman, 2017). Mistaken beliefs about synchrony’s impact on prosociality might result from biases in attention and memory: For example, synchrony may be a particularly salient form of coordinated movement, such that people notice and remember interactions involving synchronized movement more than others types of coordinated action. This would make it easier to recall instances when synchrony led to prosociality than instances when *non-synchronized* coordinated interactions led to prosociality, via an availability bias (e.g., Gilovich et al., 2002), creating the belief that synchronized actions drive prosocial behavior more than coordinated actions do.

We suspect some middle ground between these accounts to be most parsimonious. Synchrony has been reported to impact a variety of attitudinal and behavioral measures, including perceived entativity (Lakens, 2010), likeability (Hove & Risen, 2009), conformity (Wiltermuth, 2012), trust (Tamborini et al., 2018), inclusion of other in the self (Tarr et al., 2016), prejudice reduction (Atherton et al., 2019), and cooperative ability (Valdesolo et al., 2010). We hypothesize that the experience of synchrony likely impacts a smaller, more restricted range of social variables, with reported effects on some variables in the literature attributable to placebo and experimenter expectancy effects. Ultimately, identifying which (if any) of these variables are most susceptible and to what extent they are impacted by placebo and/or expectancy effects remains an open question and an important direction for future work.

In particular, the current study provided evidence that participants have expectations about prosocial attitudes – and did not measure whether these expectations extended to prosocial behaviors, like cooperation or helping. There is considerable evidence in the synchrony literature to suggest that attitudes and behaviors are linked (Jackson et al., 2018; Lang et al., 2017; Reddish et al., 2013; Tunçgenç & Cohen, 2016; Wiltermuth & Heath, 2009). The extent to which expectancy effects regarding synchrony impact prosocial behaviors is an open question, and an important direction for future work.

To determine which dependent measures are directly impacted by the experience of synchrony (versus those which reflect participants’ expectations), future work should attempt to eliminate or manipulate expectations directly. Ideally, this can be done using experimental designs where experimenters and participants are both unaware of condition. Such methods may be difficult to implement in synchrony experiments, as keeping participants naïve to the level of synchrony in their own movements while maintaining a social context may be impossible (or, at least, would require the creative use of one-way mirrors and similar experimental hijinks).

Synchrony may also be made less conspicuous by using a cover story regarding the purpose of the study, reducing the saliency of motor synchrony in the manipulation. This would require deception of both participants and experimenters, and all parties would need to be kept unaware of the motivation of the study until data collection is finished. This means that for experimenters, the cover story must be maintained over the course of data collection and be robust against experimenters’ expectations about synchrony and the true hypothesis of the study over time. If this method is used, researchers should provide periodic checks (e.g., open-ended questions) throughout data collection to ensure that the participants and experimenters have not inferred the true hypothesis. Tarr et al. (2018) used this approach, taking steps to decrease expectancy, including a cover story, a experimenter who was unaware of the hypothesis, and conducting the synchronous/asynchronous interaction in a virtual reality environment with tight control over simulated movement. Results were mixed: predicted effects of synchrony were found on only two of six measures, and most participants also did not believe their virtual partners were real, complicating interpretation.

Past work has demonstrated that the magnitude of expectancy effects may be related to the strength of participants’ expectations (i.e., a dose-dependent relationship; Sterzer et al., 2008). Thus, another potential way to account for expectancy effects is to use a control manipulation that is matched for participant expectations (Boot et al., 2013). In many cases, including the study we examine (Wiltermuth & Heath, 2009; Exp. 1), the control condition is active, and matched on many salient features (e.g., it involves taking a walk that is similar to the manipulation condition), but is not matched on participant expectations (as the results of our study show). To tease apart the effect of the intervention from the effect of expectations, a new control condition could be created that matches the experimental condition in the degree to which participants expect it will impact their response to the dependent variable or variables. Alternatively, conditions could be matched by choosing a different dependent variable—one that does not evoke differential participant expectations across conditions, but that theory predicts should be impacted by the experience of synchrony.

Achieving neutral expectations through these methods is likely to be challenging as commonly used manipulations like walking, bouncing, or hand motions may incidentally or intentionally engender participants’ expectations via awareness of synchronous movement. Fortunately, there are ways to counteract this. For instance participants’ expectations may also be experimentally manipulated, by presenting participants with evidence to change their *a priori* expectations before the task. By these methods, neutral expectancy can be achieved by presenting participants with different evidence, leading half the participants in each condition to believe that the intervention (e.g., synchronous movement) will influence their response to the dependent variable, while the other half is led to have neutral expectations (Clifasefi et al., 2006). It is also possible to explore the contributions of expectations to the effects of synchrony without creating neutral expectancy. For example, using the counterdemand design, participants could be led to believe that synchrony will only change their response to the dependent variable after a particular amount of time or exposure, and then be tested before and after this period (Boot et al., 2013).

Finally, the role of expectancy could be further tested by inducing the opposite expectation: leading participants to believe that motor synchrony *decreases* prosociality. Past research has demonstrated that conscious processes are vulnerable to expectations regardless of directionality (Benedetti et al., 2003). Thus, we believe that manipulating expectancy in the opposite direction would yield decreases in self-reported prosociality following synchrony or, at least, attenuated positive effects of synchrony. Attenuated positive effects would be expected if the expectation-change manipulation was not effective in every participant, or if expectation effects are one of multiple factors that drive synchrony’s prosocial impact. Any or all of these methods would help to determine the effects of the experience of synchrony on prosociality, over and above placebo effects, the effects of participants’ expectations.

## CONCLUSIONS

The issues we outline here threaten the validity of causal inferences in the synchrony literature, and provide an alternative causal account of how synchrony may promote prosociality (i.e., top-down expectations vs. the experience of synchrony itself). While we have not shown that no link exists between synchrony and prosociality, the existence of nuanced top-down expectations about the effect of synchrony in naïve participants suggests that experimenter bias, participant expectancy, and placebo effects likely account for some portion of a putative true effect of synchrony on prosociality. Thus, future work addressing the role of expectancy is essential for understanding the mechanism and extent to which synchrony impacts interpersonal attitudes and social behavior.

## ACKNOWLEDGMENTS

The authors thank Bronwyn Tarr and Joshua S. Bamford for valuable discussion and feedback, Michelle Lee and Jordan Legaspi for assistance with data collection, and Scott Wiltermuth for his timely assistance clarifying the method and providing original data gathered in his original publication (Wiltermuth & Heath, 2009).

## AUTHOR CONTRIBUTIONS

- Contributed to conception and design: AS, SA, & SAM
- Contributed to acquisition of data: AS, SA, & SAM
- Contributed to analysis and interpretation of data: AS, SA, & SAM
- Drafted and/or revised the article: AS, SA, & SAM
- Approved the submitted version for publication: AS, SA, & SAM

## FUNDING INFORMATION

This research was funded by the Harvard Data Science Initiative (S.A.M.), the National Institutes of Health Director’s Early Independence Award DP5OD024566 (S.A.M.), and a National Science Foundation Graduate Research Fellowship (S.A.).

## Supplemental Materials

https://github.com/themusiclab/synchrony-expectancy

https://osf.io/n5fgh
