# Supplementary material for: Expectancy Effects Threaten the Inferential Validity of Synchrony-Prosociality Research
Source: Open Mind (Camb). 2022 Dec 16;6:280–90. doi: 10.1162/opmi_a_00067 (PMC9987344; doi:10.1162/opmi_a_00067)
Supplement: Supplementary file 1 [file opmi-06-280-s001.pdf]

Supplement: Expectancy effects threaten the inferential validity of synchrony-prosociality research.

Text S1

Participant Exclusion, Sample Size, and Power Analysis

An additional 56 participants (*n* = 25 Synchrony Condition; *n* = 35 Control Condition) completed the study but took an excessively long time (> 10 minutes, >4 SD from the mean; 13 participants), failed to pass the attention check (8 participants), or failed to pass the memory check (which required them to correctly recall the instructions of the experiment, 39 participants), and were thus excluded from analysis. The study was approved by institutional review boards at both institutions, and all measures, manipulations, and exclusions are explained in detail in text or in the supplement.

We opted for an undergraduate sample in order to mimic the population used in the original study conducted by Wiltermuth & Heath (2009). The undergraduates came from both a public and a private university; moreover, the student body at [institution] is diverse in terms of both ethnicity and socioeconomic status, with approximately one-third of undergraduates the first in their family to attend a four-year college, and over 70% students of color (Clark, 2018; [institution] Institutional Research, 2020). However, the sample of undergraduates comes from two R1 research universities, limiting the diversity of our participants.

The study was preregistered, with a stopping rule of 200 participants from each institution or the end of the summer term at each location, whichever came first (the preregistration is available at https://osf.io/6g2vy/?view_only=c86f6e5d420f4ed5b151c1a1544572b3). The end of the summer term came first, at which time we had collected data from N = 216 participants. Post-hoc and

sensitivity power analyses using G*Power (Faul, Erdfelder, Buchner, & Lang, 2009) revealed that our sample size had >99% power to detect effects of a similar magnitude to those reported by Wiltermuth & Heath (e.g., d = 0.96) and a minimum effect size of interest of d = 0.29 using two-tailed two-sample independent t-tests with a Bonferroni-adjusted alpha of 0.00625 (for 8 comparisons).

Text S2

Procedure

Using instructions modeled directly on Wiltermuth and Heath's task (S. Wiltermuth, personal communication), participants were asked to imagine that three people had taken a 7-minute walk around a college campus together with an experimenter. Participants in the experimental condition were asked to imagine that people were instructed to "walk in step with one another, or in synchrony", while participants in the control read a nearly identical set of instructions that excluded the phrase that instructed people to walk in synchrony (see Supplement Text S3 for full-text for both conditions). This was the key difference between conditions, i.e., that participants in the experimental condition imagined people were instructed to complete the task in synchrony, while participants in the control condition read a nearly identical set of instructions that excluded any reference to synchrony, in a fashion identical to Wiltermuth & Heath (2009; see below for full-text of both vignettes). Participants took only a short time to read the vignettes (completion of the entire study took an average of 3 minutes and 9 seconds with a standard deviation of 1 minute and 37 seconds), consistent with the idea that they did not engage in elaborate first-person mental imagery (see Introduction).

After imagining the hypothetical scenario, participants were then asked to complete a survey where they imagined on how people would feel and act toward the other participants after engaging in the scenario they read about. This survey was modeled on the one used in Wiltermuth and Heath's original study. We included the measures of synchrony, coordination, and frustration because they were present on the survey instrument as provided by the authors (S. Wiltermuth, personal communication), though they were not reported in the original paper (Wiltermuth & Heath, 2009). Last, participants answered an attention check question (Please choose the number three; from numeric options), demographic questions (race/ethnicity, gender, age), and a memory check question.

Text S3

Vignette and Survey Full-Text

In this study, you will be asked to imagine a situation where other people participate in a live, in-person experiment. We are interested in your expectations and predictions about this situation. As you imagine it, think about what it would be like and how the people participating in the experiment might feel.

Synchrony Condition:

Imagine three college students show up to a psychology experiment here at [institution]. They are greeted by a researcher.

The researcher tells them that as part of the study, they will be taking a walk around campus — and during this walk, they will walk in step with one another, or in synchrony.
They are instructed not to talk with one another while on the walk.

The three participants and the researcher then go on a seven-minute walk around campus. They walk in step with each other the whole time.

Control Condition:

Imagine three college students show up to a psychology experiment here at [institution]. They are greeted by a researcher.

The researcher tells them that as part of the study, they will be taking a walk around campus. They are instructed not to talk with one another while on the walk.

The three participants and the researcher then go on a seven-minute walk around campus.

Afterward, the participants are asked how they feel about this experience, and about the other people in the group. Imagine how they might answer as you respond to the questions below.

Presented on a subsequent page:

As you respond to the questions below, we will ask you to judge how the three participants in the scenario might feel about one another after this experience.

Remember: In the scenario you read, three college students showed up for a psychology study, and were asked to [walk around campus with one another] / [walk around campus in step with one another, in synchrony].

Please imagine how these people might feel after this experience, as you answer the questions below.

1. **How coordinated** would each participant's actions be with the other participants on the walk?

Not Coordinated 1 2 3 4 5 6 7 Very Coordinated

2. To what degree would each participant feel they were **on the same team** with the other participants during the walk?

Not At All 1 2 3 4 5 6 7 Very Much

3. **How connected** would each participant feel with the other participants on the walk?

Not At All 1 2 3 4 5 6 7 Very Much

4. How much would each participant **trust** the other participants?

Not At All 1 2 3 4 5 6 7 Very Much

5. **How similar** would each participant feel to the other participants?

Not Similar 1 2 3 4 5 6 7 Very Similar

6. **How happy** would each participant feel?

Not Happy 1 2 3 4 5 6 7 Very Happy

7. **How frustrated** would each participant feel?

Not Frustrated	1	2	3	4	5	6	7	Very Frustrated

8. **How synchronized** would each participant's actions be with those of the other participants on the walk? In other words, how much would each participant move in time with the other participants during the walk?

Not Synchronized	1	2	3	4	5	6	7	Very Synchronized

9. Please choose the number **three** from the options below.

	1	2	3	4	5	6	7	

Presented on a subsequent page:

What is your **race**?

- American Indian/ Alaska Native
- Asian
- Native Hawaiian or Other Pacific Islander
- White
- More than One Race
- Prefer not to say

Are you **Hispanic or Latino**?

- Yes
- No
- Prefer not to say

Think about the scenario you read earlier. **What instructions** did the researcher give the students?

- Take a walk around campus. Get to know each other while on the walk.
- Take a walk around campus. Do not talk with one another.
- Take a walk around campus, and walk in synchrony. Get to know each other while on the walk.
- Take a walk around campus, and walk in synchrony. Do not talk with one another.

What is your **gender**?

- Male
- Female
- Other__________

What is your **age** in years? _________

Results

Table S1

Means, Standard Deviations, t-statistics, and p-values for all comparisons of predicted feelings

Predicted Feeling	Imagined Synchrony		Imagined Control		$t(214)$	p	*Cohen's d*
	M	*SD*	*M*	*SD*			
Connection	4.42	1.49	2.97	1.51	7.10	< .00625	.97
Trust	3.76	1.48	2.82	1.28	5.00	< .00625	.68
On the same team	5.50	1.39	3.92	1.67	7.55	< .00625	1.03
Similarity	4.62	1.46	4.07	1.69	2.55	< .05	.35
Happiness	3.85	1.12	3.64	1.12	1.37	.17	.19
Coordination	5.10	1.41	4.08	1.46	5.21	< .00625	.71
Synchrony	5.22	1.31	4.34	1.52	4.57	< .00625	.62
Frustration	3.66	1.46	3.72	1.61	0.28	.78	.04

Figure S2

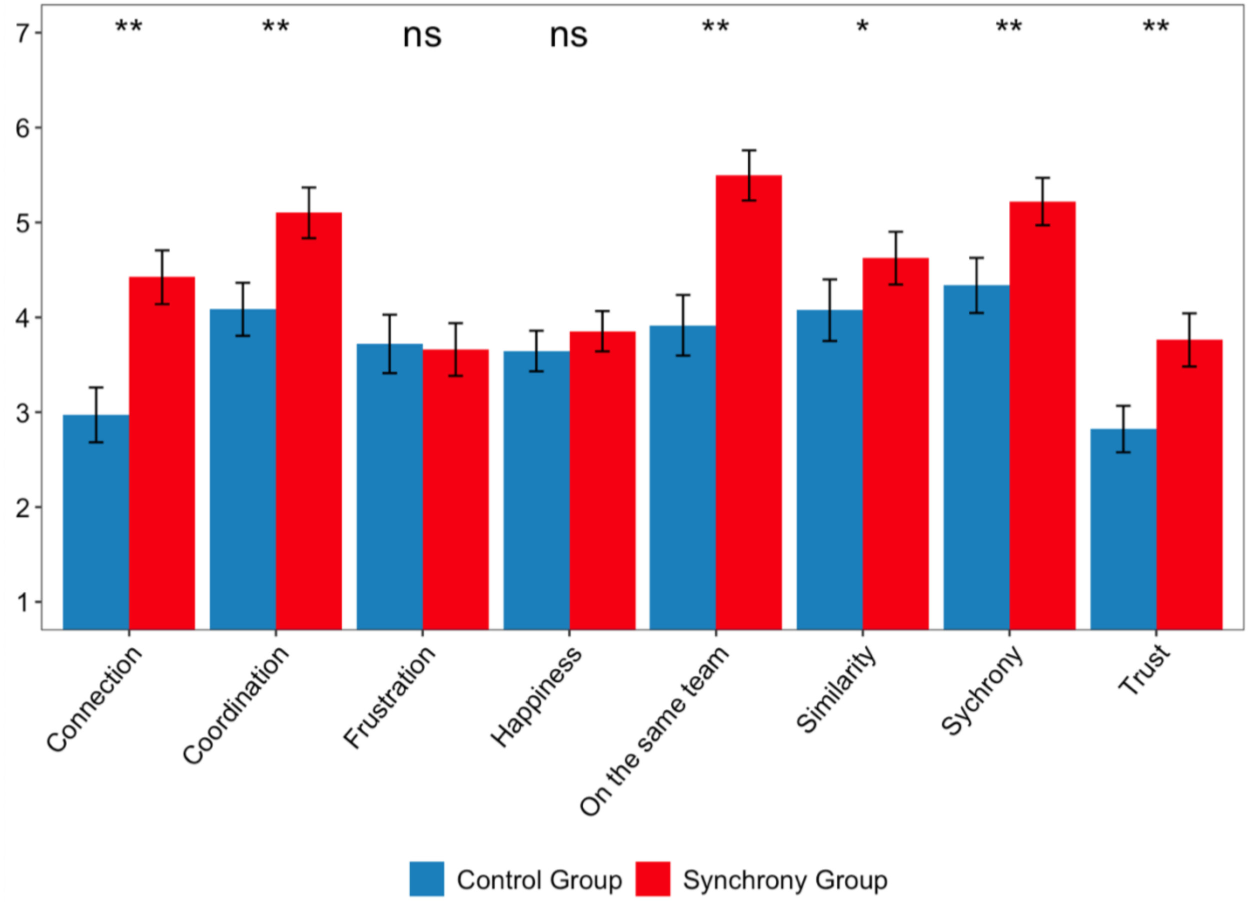

Figure S2. Participants' expectations about people's attitudes and feelings after engaging in synchrony or control. This supplemental figure includes results for synchrony, coordination, and frustration, measures that were not reported in the paper by Wiltermuth & Heath (2009), but were included in a survey instrument supplied by the authors (S. Wiltermuth, personal communication). Participants were asked to rate on a scale from 1 to 7 how connected, coordinated, frustrated, and so on (*x*-axis) they imagined people would feel, if they had just walked around campus in synchrony (red bars) or in a normal manner, with no mention of synchrony (blue bars) with their group. Error bars represent 95% confidence intervals. $^{**}p < .00625$, $^{*}p < .05$, from two-sample independent t-tests.

Appendix S3

Full regression tables predicting overlapping variables between current study and Wiltermuth and Heath, 2009 Study 1 from study type (imagined, in the current study; vs. experienced, in the Wiltermuth & Heath study), condition (synchrony vs. control/neutral), and their interaction.

Predicting Amount of Connection from Condition & Study Type

Term	$\hat{\beta}$	Std. Error	t	p
Intercept	2.93	0.39	7.47	<.001
Condition	1.60	0.56	2.88	.004
Study Type	0.04	0.42	0.09	.927
Condition × Study Type	-0.15	0.59	-0.25	.801

Predicting Happiness from Condition & Study Type

Term	$\hat{\beta}$	Std. Error	t	p
Intercept	4.80	0.29	16.44	<.001
Condition	-0.07	0.41	-0.16	.872
Study Type	-1.16	0.31	-3.71	<.001
Condition × Study Type	0.28	0.44	0.62	.533

Predicting Feelings of Being on the Same Team from Condition & Study Type

Term	$\hat{\beta}$	Std. Error	t	p
Intercept	3.73	0.41	9.18	<.001
Condition	1.27	0.58	2.20	.029
Study Type	0.18	0.43	0.42	.675
Condition × Study Type	0.31	0.61	0.51	.611

Predicting Perceptions of Similarity from Condition & Study Type

Term	$\hat{\beta}$	Std. Error	t	p
Intercept	3.13	0.40	7.93	<.001

Condition	1.33	0.56	2.39	.018
Study Type	0.94	0.42	2.23	.027
Condition × Study Type	-0.78	0.60	-1.32	.190

Predicting Level of Synchrony from Condition & Study Type

Term	$\hat{\beta}$	Std. Error	t	p
Intercept	3.60	0.37	9.86	<.001
Condition	2.40	0.52	4.65	<.001
Study Type	0.74	0.39	1.89	.060
Condition × Study Type	-1.52	0.55	-2.75	.006

Predicting Amount of Trust from Condition & Study Type

Term	$\hat{\beta}$	Std. Error	t	p
Intercept	4.07	0.36	11.39	<.001
Condition	1.53	0.51	3.03	.003
Study Type	-1.24	0.38	-3.26	.001
Condition × Study Type	-0.59	0.54	-1.10	.272
